# Up is best

**DOI:** 10.1177/20416695231190236

**Published:** 2023-08-05

**Authors:** Stuart Anstis, Patrick Cavanagh

**Affiliations:** Department of Psychology, University of California San Diego, La Jolla, USA; Department of Psychology, Glendon College, North York, ON, Canada; Centre for Vision Research, York University, North York, ON, Canada

**Keywords:** contours/surfaces, scene perception, shapes/objects, spatial cognition

## Abstract

Ambiguous patterns have a tendency to appear to point up. This bias makes sense as most objects are on the ground, pointing up. However, we discover that the source of the up bias is the preference for seeing depth receding from the lower to the upper visual field.

Figure 1 shows horizontal rows of spikes, random in the top two panels and regular in the bottom two. On the left, each row is progressively lighter moving down while on the right, each row gets progressively darker. These patterns reveal a strong perceptual bias. The patterns are fully ambiguous with respect to direction, so one might expect the spikes to be seen equally often pointing up or pointing down or to be pointing up in some regions and down in others. But we found a strong “up” bias. Observers who viewed Figure 1 at a vision conference interpreted the spikes as pointing up ∼ 70% of the time where chance would be 50%.

**Figure 1.**
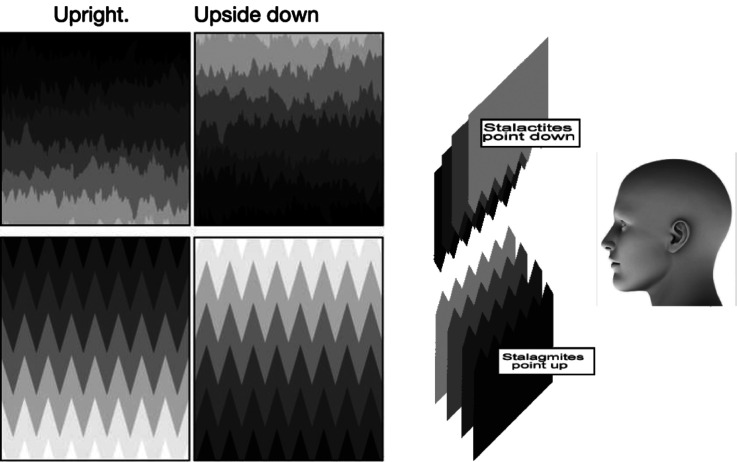
The spikes in all four patterns on the left tend to be seen as pointing up, as depicted on the right.

What can explain this strong “Up” preference? Nearly everything we see stands on the ground pointing UP (people, trees, houses, and telephone poles). Hanging objects that point DOWN (crane hooks, hanging lamps, and sleeping bats) are rare (Figure 2). So, our perceptual diet may bias our orientation assumptions. This bias toward pointing up is comparable to the well-known bias toward seeing light from above in our sunlit world (Adams, 2007; Mamassian & Landy, 2001).

**Figure 2.**
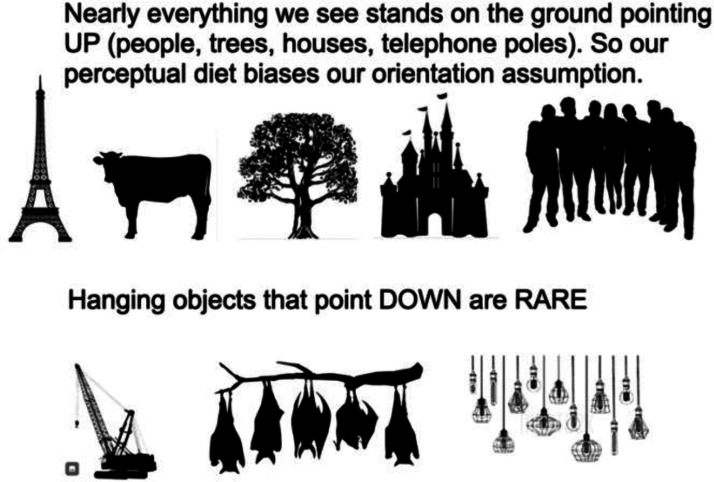
Standing things are more common than hanging things.

However, the biases we find in the images of Figure 1 may have another source. The lightness gradient appears to create a stepped series of layers stacked in depth like a stack of papers of different gray levels that cover one another. When the *bottom* row looks nearest in-depth, it asserts ownership of its upper jagged edge, and this edge, therefore, appears to point up and cover the row above it. If instead, the *top* row were to look nearer in depth, it would assert ownership of its lower jagged edge. This edge would then appear to point down and each row would cover the row below it. Importantly, most observers see these rows as stepping backward in-depth, closer to the bottom than the top.

Is it the lightness gradients that are producing the depth order? In Figure 3, we see that removing the lightness gradient removes both the perceived depth and the perceived pointing bias. Coloring the rows of triangular or random spikes from Figure 1 with random grays makes the rows look like horizontal zigzag lines whose edges point neither up nor down, or alternately either, and they also lose the impression of depth, becoming flat two-dimensional textures. Each jagged edge simply divides the adjacent strips without being owned by either, thus there is no sense that one strip is occluding the next strip above it or below it and continuing behind it. This suggests that the depth order must come first, and it then determines the pointing bias. So, for example, it could be that the bottom rows in the images of Figure 1 are preferentially seen as nearest because they represent the ground plane.

**Figure 3.**
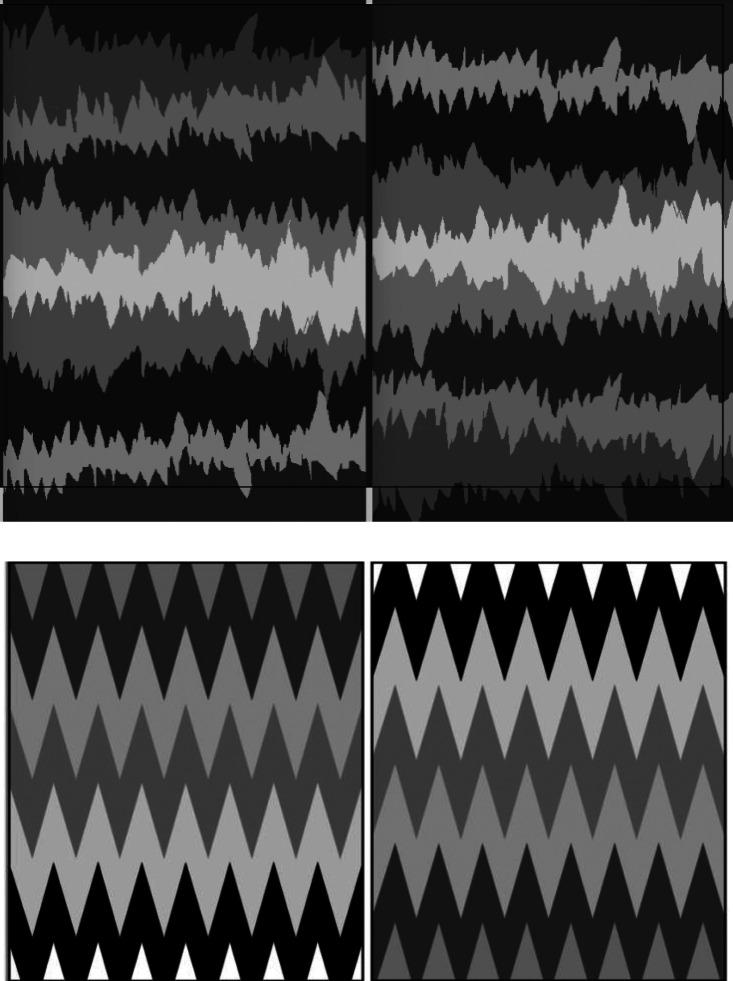
When the gray levels in Figure 1 are randomized, the perceived depth ordering and upward pointing bias both disappear.

As a test, Figure 4 presents stereo pairs that can produce a depth order that drives pointing directions. Free-fuse either the leftmost pair or the rightmost pair in Figure 4 to produce rows that are either closest at the top or at the bottom. In the stereo pair where the bottom row looks nearest, it (and every other row) will assert ownership of its top edge, and each edge will appear to point up. In the other stereo pair, where the top row looks nearest, it (and every other row) will assert ownership of its bottom edge, which will appear to point down. However, this latter case is unstable and sometimes the spikes will appear to point up in defiance of the stereo cues. The outcome is the same if the gray gradient has white at the top instead of at the bottom, as one can confirm by turning the image upside down.

**Figure 4.**
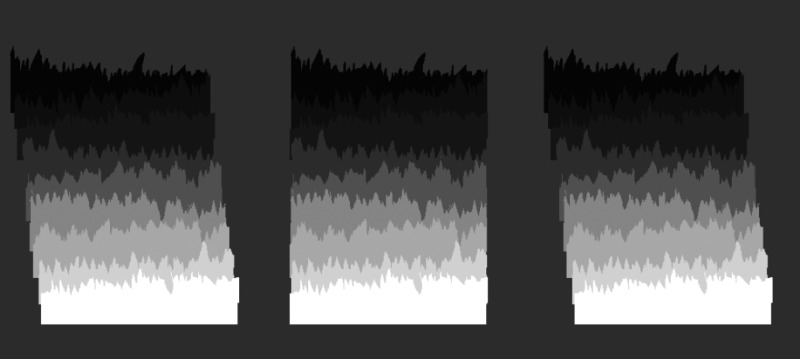
The left-hand two images can be free-fused to produce rows that are staggered back in depth, being closest at the top (convergent free fusing) or closest at the bottom (divergent). The depth is reversed when free-fusing the right-hand two images.

These observations indicate that the depth constrains border ownership, which causes the spikes to point apparently up or down. In this sequence, depth → border ownership → spike directions, so the pointing bias is an effect not a primary cause. When the stereo depth imposes bottom-near, it reinforces the up-bias and all spikes point upwards. When depth imposes top-near it goes against the up-bias and spikes point mostly down but also waver towards pointing up.

The pointing and depth order biases are head-centric, not earth-centric, so they rotate with your head. Take a pattern of spikes that appear to point upwards, namely toward your forehead and towards the ceiling. Now turn your head upside down and look at the pattern. The spikes still appear to point “up,” towards your forehead but now towards the floor.

In conclusion, the up bias is caused principally by a depth bias: objects at our feet are closer and lower in the field of vision than those at a distance. This well-known, height-in-field effect imposes a depth order and an upward direction on otherwise ambiguous patterns, as long as there are image cues that support a depth ordering. In the examples we presented, the height-in-field effect required a luminance gradient from near to far to allow a depth interpretation but, interestingly, the gradient could be in either direction. Lighter on top is consistent with atmospheric perspective and lighter on the bottom is consistent with an illumination source that is near the observer. Although most objects in our experience are standing on the ground and pointing up, it is not this factor that dominates in producing the up bias, it is the bias to see depth receding with increasing height in the field that imposes the border ownership that drives the up bias.
